# Supplementary figures and images for: Identification of QTLs with effects on seed coat appearance in cowpea
Source: PLoS One. 2025 Oct 8;20(10):e0333353. doi: 10.1371/journal.pone.0333353 (PMC12507197; doi:10.1371/journal.pone.0333353)

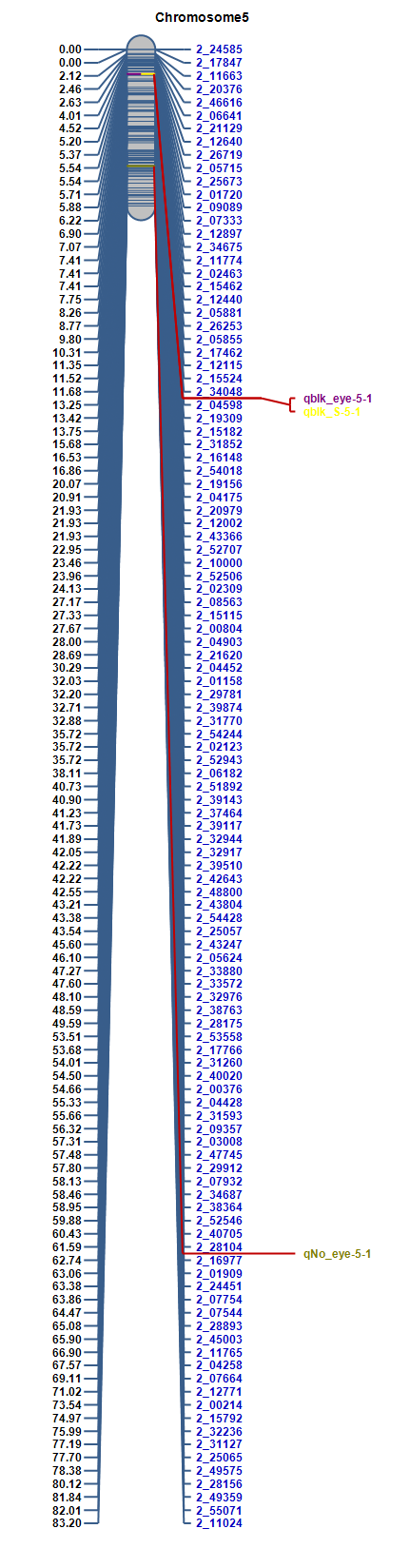

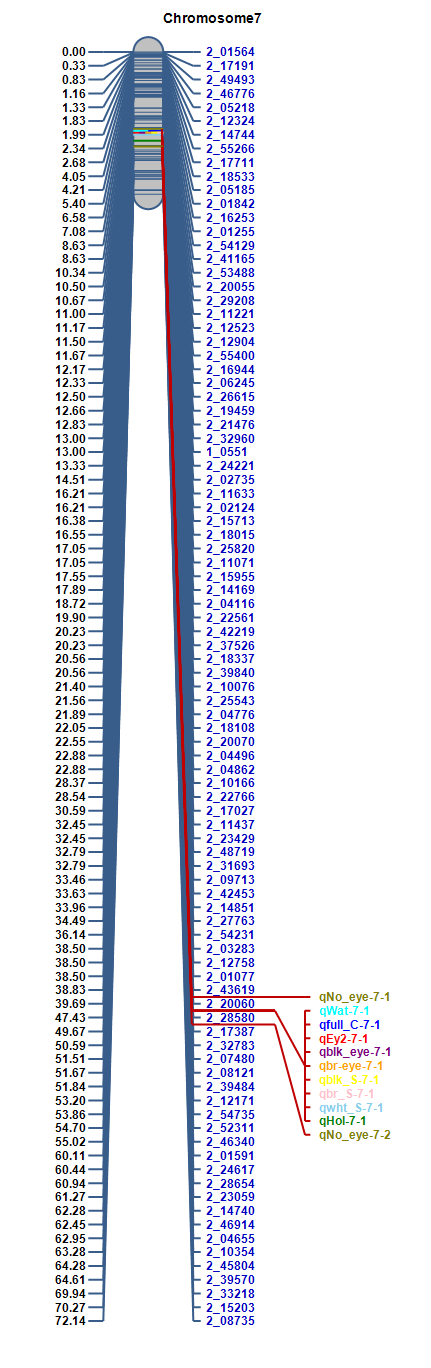

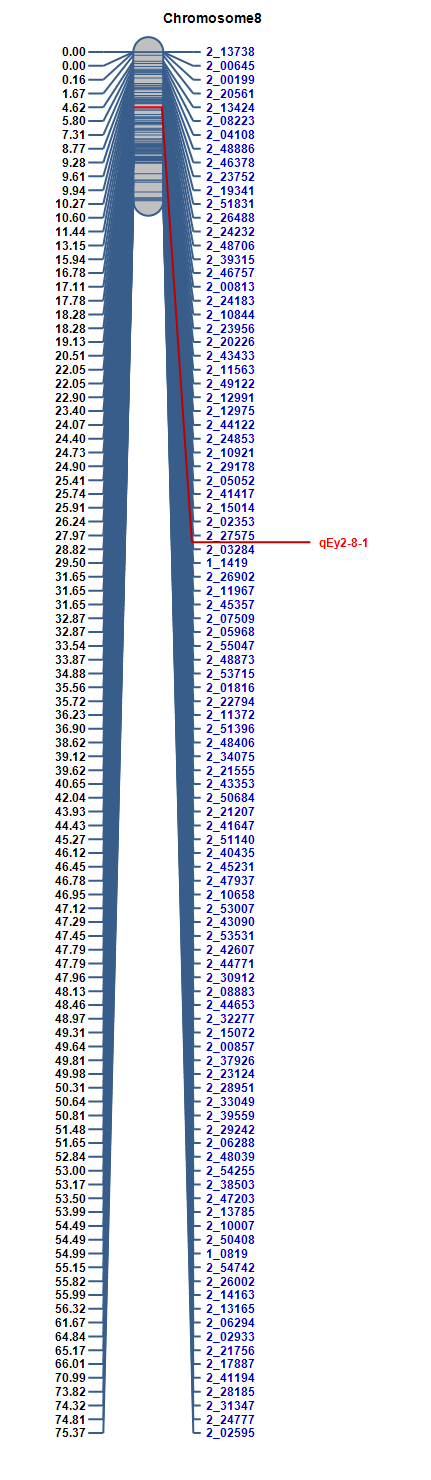

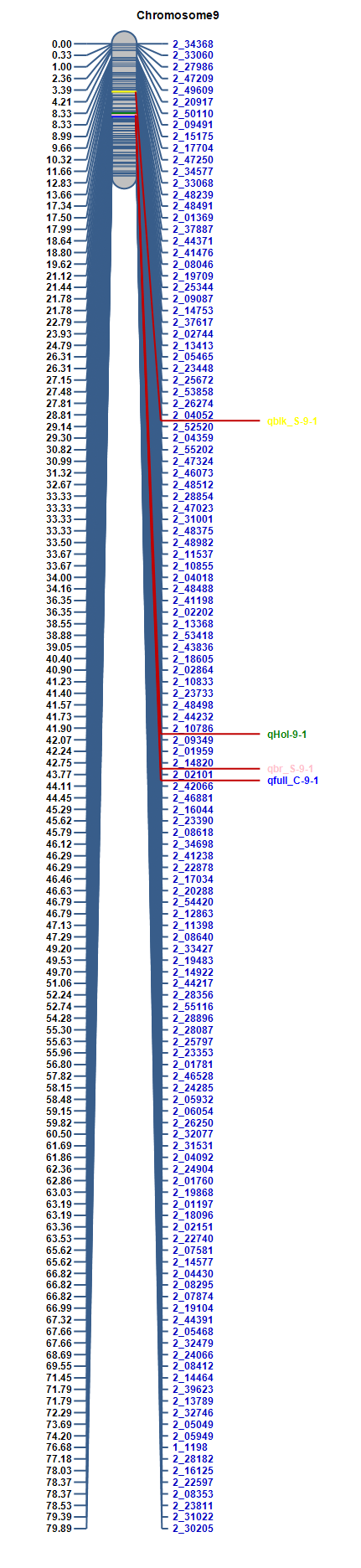

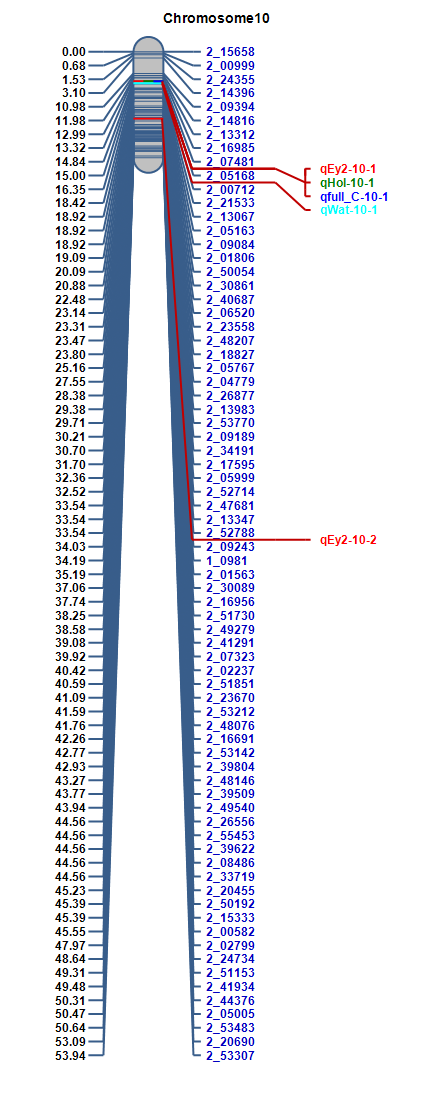

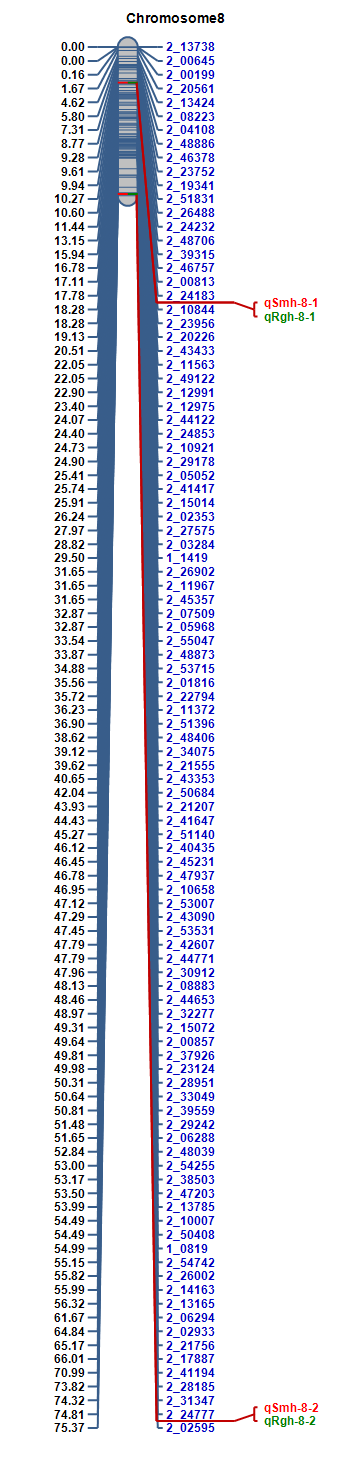

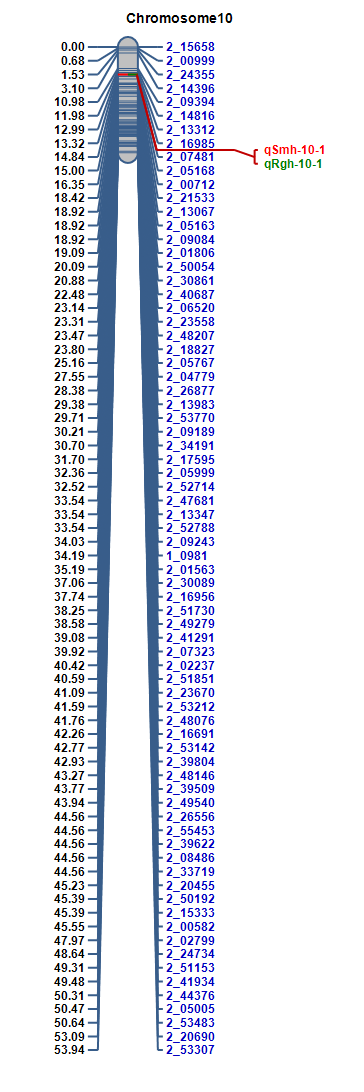


**Figure S1**. QTLs positions of seed coat traits (A) Seed coat colour and pattern (B) Seed coat texture

Supplement: S1 Fig — (DOCX) [file pone.0333353.s001.docx]
