# Supplementary material for: Identification of QTLs with effects on seed coat appearance in cowpea
Source: PLoS One. 2025 Oct 8;20(10):e0333353. doi: 10.1371/journal.pone.0333353 (PMC12507197; doi:10.1371/journal.pone.0333353)
Supplement: Attachment — Submitted filename: Report (2).docx [file pone.0333353.s007.docx]

**Dear Editor,**

**Reviewer Report**

**Title:** Genetic Dissection of Seed Coat Pigmentation in Cowpea (Vigna unguiculata [L.] Walp.): Identification of QTLs and Epistatic Interactions Using a RIL Population

**1. General Comments**

The manuscript presents a comprehensive QTL mapping study on cowpea seed coat pigmentation using a recombinant inbred line (RIL) population. The study integrates genotypic data from a mid-density DArTag SNP panel and phenotypic evaluations conducted over two years. It provides valuable insights into the complex inheritance of seed coat traits, identifies both main-effect and epistatic QTLs, and proposes several biologically relevant candidate genes based on functional annotation.

The work is timely and relevant, especially given the rising demand for legume varieties that meet market and consumer preferences. The use of high-resolution SNP genotyping and epistatic QTL analysis is commendable, and the findings hold direct implications for cowpea breeding programs targeting seed quality traits.

**2. Strengths**

- **Relevance and Novelty:** Seed coat pigmentation is a key market trait in cowpea. This study fills a gap by exploring its genetic architecture using a biparental population.
- **Robust Experimental Design:** The use of 316 RILs evaluated over two seasons under field conditions adds strength to the phenotypic dataset.
- **High-Density Genotyping:** The use of 2602 high-quality SNPs across the genome improves QTL detection power.
- **Integration of Epistasis Analysis:** The identification of 116 digenic epistatic interactions offers insights into the complex inheritance beyond single-locus effects.
- **Biological Interpretation:** The identification of 12 putative candidate genes enhances the translational value of the findings for marker-assisted selection and gene cloning.

**3. Weaknesses and Suggestions for Improvement**

**a) Clarify Seed Coat Trait Definitions**

- Provide more detail on how seed coat pigmentation and texture were scored. Include whether digital imaging, colorimetric scores, or visual ratings were used.

**b) Statistical Thresholds and Methods**

- Clearly state the statistical models, QTL significance thresholds (e.g., LOD cutoff), and software used (e.g., R/qtl, QTL IciMapping, TASSEL). If permutation tests were used, please report how many iterations were performed.

**c) Effect Sizes of QTLs**

- Although QTLs with PVE (phenotypic variance explained) are reported, the manuscript should emphasize which QTLs are stable and robust across years/environments.

**d) Candidate Gene Annotations**

- A table summarizing the 12 candidate genes with gene IDs, putative functions, chromosomal location, and references will strengthen the biological relevance. Consider using functional enrichment analysis or expression data if available.

**e) Epistasis Interpretation**

- Provide a figure or matrix summarizing epistatic QTL pairs and highlight any biologically meaningful interactions. Discuss the potential breeding implications of these interactions, even if minor.

**f) Language and Technical Corrections**

- Improve grammatical clarity throughout. For example:
  - "mapbased cloning" → "map-based cloning"
  - "quality DArTag single nucleotide polymorphisms (SNPs)" → consider simplifying to "a panel of 2602 high-quality DArTag SNPs"

**4. Minor Suggestions**

- Include a figure showing representative seed coat patterns of parental lines (RP270 and CB27) and selected RILs.
- Ensure consistent usage of units, e.g., percentages for PVE.
- All abbreviations (e.g., QTL, SNP, RIL) should be defined at first use in the manuscript.

**5. Recommendation**

**Minor Revision**

The manuscript presents a well-structured and scientifically valuable contribution to cowpea genetics and breeding. After addressing the points listed above—especially improved clarity on trait phenotyping, candidate gene presentation, and analytical methods—the study will be suitable for publication.

Sincerely,

Muraleedhar S. Aski
