# Supplementary material for: Identification of QTLs with effects on seed coat appearance in cowpea
Source: PLoS One. 2025 Oct 8;20(10):e0333353. doi: 10.1371/journal.pone.0333353 (PMC12507197; doi:10.1371/journal.pone.0333353)
Supplement: Attachment — Submitted filename: Response to Reviewers.docx [file pone.0333353.s010.docx]

Dear Editors-in-Chief,

I am writing to submit the revised version of the manuscript titled "***Identification of QTLs with effects on seed coat appearance in cowpea*"** following reviewers’ comments and suggestions received from PLOS ONE Journal

We really appreciate the editor and the reviewers’ positive and insightful comments and suggestions, which have enabled us to improve the write-up. Below, we provide a point-by-point response to each reviewer’s comment. All changes have been incorporated into the revised version of the manuscript, with track changes for clarity.

**i.	Editor requests**

**- The role of the funder:** The funder of the study reported in this manuscript, the Accelerated Varietal Improvement and Seed Delivery of Legumes and Dryland Cereals in Africa (AVISA), had no role in the study design, data collection and analysis, decision to publish, or preparation of the manuscript.

**i.	Editor’s requests**

**- The role of the funder:** "The funder ~~s~~ of the study reported in this manuscript, the Accelerated Varietal Improvement and Seed Delivery of Legumes and Dryland Cereals in Africa (AVISA), had no role in the study design, data collection and analysis, decision to publish, or preparation of the manuscript.

**- Laboratory protocols**

We appreciate the editor's comments that we should submit the laboratory protocols to support reproducibility. However, in our case, this recommendation is not applicable. We did not perform laboratory-based DNA extraction or genotyping ourselves; the leaf samples were collected and prepared in accordance with the standard procedures required by the genotyping company (Intertek Agritech laboratory) as described in the Materials and Methods section. We have ensured that all steps under our control, especially the sampling procedures, data collection, and data analyses, are thoroughly described in the manuscript to allow reproducibility.

**- Data availability**

We really acknowledge PLOS ONE’s policy on open data sharing to allow transparency and reproducibility. The phenotypic data and all QTL results generated in the present study have been made available in the manuscript and supporting information. The genotypic data used are currently part of a larger ongoing research project and can be accessed by contacting the principal investigator at [O.Boukar@cgiar.org](mailto:O.Boukar@cgiar.org) and eventually will become available through the IITA open access database at https://data.iita.org/.

**ii.	Reviewer 1’^s^ comments**

a) **Clarify Seed Coat Trait Definitions**

We have revised the Materials and Methods section (highlighted area in lines 127-129) to provide a clearer description of the scoring of the seed coat pigmentation and seed coat texture.

b) **Statistical Thresholds and Methods**

We have revised the Materials and Methods section (in lines 158-161) to provide detailed information on the QTL mapping. QTL analysis was conducted using QTL IciMapping version 4.2. For both main-effect and epistatic QTL detection, we performed permutation tests with 1000 iterations to determine appropriate LOD thresholds for significance, as estimated by the software.

c) **Effect Sizes of QTLs**

We really appreciate the reviewer’s suggestion on stable QTL. However, we did not carry out separate QTL analyses for each year because the phenotypic data collected across the two seasons were highly consistent, reflecting the genetic stability of the RIL population used. As a result, we performed a QTL analysis using the combined data from both years that enabled us to increase the statistical power and detect QTLs that represent the overall genetic effects across years. However, in response to the reviewer’s comment, we have now conducted separate QTL analyses for each year (S3 Table). The results confirm that the major QTLs identified in the combined analysis are also consistently detected in both individual years.

d) **Candidate Gene Annotations**

We have added a new table (Table 3) in Lines-287-289 summarizing the candidate genes, including gene IDs, chromosomal number, predicted functions, and supporting references.

e) **Epistasis Interpretation**

We appreciate the reviewer’s insightful comment on the importance of visualizing and interpreting epistatic interactions. In the original submission (Figure 4) is provided, that shows the digenic epistatic QTL interactions. This figure presents the significant epistatic QTL pairs detected across the 11 chromosomes of cowpea and highlights potential functional interactions relevant to seed coat pigmentation. To further strengthen the biological relevance of these findings, we have now provided a minor discussion on the potential breeding implications of these epistatic interactions in the revised manuscript (Discussion section, from lines 376 to 380).

**Minor Suggestions**

Regarding the inclusion of a figure showing representative seed coat patterns of parental lines (RP270 and CB27) and selected RILs, we would like to kindly note that this figure was already included in the initial submission as Figure 1. It presents the seed coat patterns of the two parental lines (RP270 and CB27) alongside selected RILs.

We have checked the manuscript and ensured that all abbreviations are clearly defined upon their first mention.

**Reviewer 2’^s^ comments**

We thank the reviewer for his suggestions, and the following details have been added from lines 261 to 263:

- We added the appropriate reference for the *Vigna unguiculata* (IT97K-499-35) in lines 167-168.

- The genes were retrieved from the Phytozome v1.1 database (http://phytozome.jgi.doe.gov/), and the InterPro portal was used for gene models along with their functional annotations.

- For the flavonoid biosynthesis pathway, no original expression data were generated in this study; we only used expression pattern references where available.

- We have revised Supplementary Tables S2 and S3 and increased the figure resolution.

- The names of the QTLs, chromosome numbers, and connecting lines in Figure 3 are fully visible when the PDF version of the manuscript is zoomed in, as the figures were submitted in high resolution.

The manuscript has been thoroughly revised and agreed upon by all authors.

Thank you for your time and consideration. We look forward to hearing from you soon.

Sincerely,

Abdoul Moumouni Iro Sodo

International Institute of Tropical Agriculture

abdoulmoumouniirosodo@gmail.com
