# Supplementary material for: Identification of QTLs with effects on seed coat appearance in cowpea
Source: PLoS One. 2025 Oct 8;20(10):e0333353. doi: 10.1371/journal.pone.0333353 (PMC12507197; doi:10.1371/journal.pone.0333353)
Supplement: Attachment — Submitted filename: Response_to_Reviewers_auresp_2.docx [file pone.0333353.s011.docx]

Dear Editors-in-Chief,

I am writing to submit the revised version of the manuscript titled "***Identification of QTLs with effects on seed coat appearance in cowpea*"** following reviewers’ comments and suggestions received from PLOS ONE Journal

We really appreciate the editor and the reviewers’ insightful comments and suggestions, which added value of the manuscript. Below, we provide a point-by-point response to each reviewer’s comment. All changes have been incorporated into the revised version of the manuscript, with track changes for clarity.

**i.	Editor requests**

We are grateful for insightful comments. We have crosschecked and reviewed the reference list and confirmed that it is complete, correctly formatted, and that none of the cited reports has been retracted and no additional citation was requested by the reviewers.

**Reviewer^s’^ comments**

- **Length and density of abstract.**

Comment: Slightly word-heavy for an abstract; some sentences could be shortened for more impact. High number of numeric details in one paragraph can overwhelm the reader.

Response: We appreciate the observation and have condensed the abstract by merging related sentences (Line14-line16) and (line 20-line21).

- **Lack of novelty emphasis**

Comment: Does not explicitly highlight what is new compared to previous studies on cowpea pigmentation QTLs. One major question: "What is the advancement beyond earlier mapping studies?" please mention.

Response: We appreciate the comment. We have emphasized that our study reports the first mapping of QTLs for seed coat texture in cowpea in the introduction indicating (line 94-line 96). For Identification of novel, to address this, we have now added a new Table 4 (line 309-line 317) comparing the QTLs identified in this study with those reported previously in cowpea seed coat appearance studies. These comparative highlights that 14 out of the 30 QTLs detected are novel, providing strong evidence of advancement beyond earlier mapping efforts and the integration of epistatic QTL analysis, revealing 116 significant interactions, which has rarely been done for these traits in cowpea (Line 266-line 269).

- **Limited statistical clarity**

Comment: The term “major QTLs” is used, but the LOD thresholds, confidence intervals, or effect size definitions are not given (though some of this may be fine to omit for space). “Much smaller effect” for epistatic QTLs is qualitative; could be backed with a range or average.

Response: We appreciate the comment, and we now define major QTLs as those explaining ≥10% phenotypic variance (PVE) in methodology (line 162-line 164) as per standard QTL mapping threshold. In the Results section, we provide average and range of PVE for epistatic QTLs (line 268-line 270).

**Trait definition could be sharper**

Comment: “Seed coat appearance traits” is broad—could briefly define if this includes coat color, eye pattern, mottling, etc.

Response: We agree and have defined early in the Methodology (line123-126) that seed coat appearance traits in present study include seed coat color, seed eye color, seed coat color pattern, and seed coat texture.

Comment: Currently, pigmentation focus is clear, but texture (mentioned in the intro) is not addressed in the results.

Response: Thank you for the observation; the seed texture has been addressed; the QTL for texture is presented in Table 2 and the texts in lines 248-249 specifies the number of QTLs identified for seed coat texture.

**Candidate gene evidence**

Comment: Mentions 12 candidate genes from GO/literature but does not say whether any are novel or validated. Lacks mention of functional categories (e.g., flavonoid biosynthesis, anthocyanin pathway) that could make it more engaging.

Response: We have grouped the 12 candidate genes into three functional categories flavonoid biosynthesis, transcriptional regulation, and ubiquitin-mediated regulation and indicated which genes are novel in cowpea pigmentation studies (lines-440-444).

**Marker-assisted selection (MAS) potential**

Comment: While breeding application is stated, the potential for predictive accuracy or MAS efficiency is not quantified.

Response: Thank you for this insightful observation: In response, we have now conducted a Relative Efficiency of Marker-Assisted Selection (RE-MAS) analysis for the major QTLs identified in this study showing the comparative efficiency of MAS relative to phenotypic selection (Line 170-Lines 189). These results provide a quantitative assessment of the breeding value of the identified QTLs and strengthen the practical application of our findings for marker-assisted selection in cowpea improvement Table 5 in lines 318-331.

The discussion section has also been updated to highlight the implications of these results for breeding programs (lines 445-455).

The manuscript has been thoroughly revised and agreed upon by all authors.

Thank you for your time and consideration. We look forward to hearing from you soon.

Sincerely,

Abdoul Moumouni Iro Sodo

International Institute of Tropical Agriculture

abdoulmoumouniirosodo@gmail.com
